# Artificial Intelligence Integration in Radiology in Saudi Arabia: A Cross-Sectional Survey of Workforce Readiness and Implementation Challenges

**DOI:** 10.3390/healthcare14172711

**Published:** 2026-08-25

**Authors:** Abdullah O. Alamoudi, Yousif M. Abdallah

**Affiliations:** Department of Radiological Sciences and Medical Imaging, College of Applied Medical Sciences, Majmaah University, Majmaah 11952, Saudi Arabia

**Keywords:** artificial intelligence, radiology, diagnostic imaging, surveys and questionnaires, Saudi Arabia

## Abstract

**Highlights:**

**What are the main findings?**

- Participants showed favorable attitudes toward AI (3.57 ± 0.69) but lower implementation readiness (3.04 ± 0.79).
- Perceived barriers were substantial (3.67 ± 0.64), particularly implementation cost and limited digital infrastructure.
- Insufficient staff training and lack of technical expertise were also major implementation challenges.
- AI knowledge was positively associated with attitudes (r = 0.49) and implementation readiness (r = 0.39).
- Attitudes were positively associated with implementation readiness (r = 0.44).
- No significant differences were observed among professional groups (all *p* > 0.05).

**What are the implications of the main findings?**

- Positive attitudes toward AI do not necessarily indicate institutional readiness for implementation.
- Investment in digital infrastructure and technical capacity should accompany AI adoption.
- Role-specific AI education and workforce training should be prioritized.
- Multidisciplinary governance addressing privacy, ethics, cybersecurity, and regulation is needed.
- AI should be introduced through phased, monitored implementation programs.
- Future studies should directly evaluate clinical, economic, workflow, resource-use, and environmental outcomes.

**Abstract:**

**Background:** Artificial intelligence (AI) is increasingly being integrated into radiology and may improve diagnostic efficiency and workflows. Its successful adoption depends on workforce readiness, organizational capacity, and effective governance. This study evaluated radiology professionals’ perceptions of AI integration in Saudi Arabia; sustainability was considered only as a conceptual implementation context and was not directly measured. **Methods:** An online cross-sectional survey using convenience sampling was conducted among healthcare professionals involved in radiology services in Saudi Arabia, using an expert-reviewed and pilot-tested 30-item questionnaire covering knowledge, attitudes, implementation readiness, and perceived barriers. Responses from 295 healthcare professionals were analyzed using descriptive statistics, Cronbach’s alpha, Spearman correlation, and Kruskal–Wallis testing. **Results:** Participants demonstrated positive attitudes toward AI integration (3.57 ± 0.69) and moderate knowledge (3.35 ± 0.70), whereas implementation readiness was comparatively lower (3.04 ± 0.79). Perceived barriers showed the highest domain score (3.67 ± 0.64). Major barriers included implementation costs (3.98 ± 0.71), limited digital infrastructure (3.90 ± 0.75), insufficient staff training (3.84 ± 0.77), and lack of technical expertise (3.82 ± 0.78). Positive correlations between knowledge, attitudes, and implementation readiness were observed. **Conclusions:** Among participating respondents, support for AI integration was generally favorable, but lower perceived institutional readiness and organizational, infrastructural, and governance barriers may constrain implementation. Because the convenience sample lacked a sampling frame and a calculable response rate, the results should not be interpreted as national estimates. The survey also did not measure clinical, economic, resource-use, or environmental outcomes and therefore does not establish sustainability benefits. Future probability-based and implementation studies should evaluate representativeness and these outcomes directly.

## 1. Introduction

Artificial intelligence (AI) has emerged as one of the most transformative technologies in healthcare, with radiology representing one of its earliest and most mature areas of clinical implementation [1,2,3]. AI applications, including machine learning and deep learning algorithms, have demonstrated considerable potential to assist image interpretation, automate repetitive tasks, optimize workflow management, improve quality assurance, and support clinical decision making, thereby enhancing diagnostic efficiency and healthcare service delivery [4,5,6,7,8,9,10,11,12,13,14,15,16]. However, successful implementation depends not only on algorithmic performance but also on organizational readiness, workforce capability, governance, and effective integration into routine clinical workflows [3,4,5,6,15,16,17,18,19,20,21,22,23].

Sustainability is an important consideration in digital-health transformation and may encompass environmental performance, resource utilization, economic resilience, workforce development, governance, and equitable access [1,2,3,4,5,6,7,8,9,10,24,25,26]. In radiology, AI has been proposed as a potential influence on some of these dimensions through changes in workflow and resource use. These propositions describe possible downstream effects, however, and are not outcomes captured by a workforce perception survey [27,28,29].

Existing literature identifies both potential benefits and potential environmental costs of AI. Workflow optimization might reduce unnecessary imaging or improve equipment utilization, whereas model development, deployment, data storage, and computation consume energy [7,8,9,10,30,31,32,33,34,35]. The net sustainability effect therefore depends on the technology, infrastructure, energy sources, and implementation choices and must be established using clinical, operational, economic, and environmental indicators. In the present study, this literature provided conceptual context only; no energy, emissions, resource-consumption, cost-effectiveness, access, or patient-outcome measures were collected.

Successful AI implementation therefore requires a comprehensive sociotechnical approach extending beyond technological capability. Previous research has consistently identified digital infrastructure, interoperability, governance, cybersecurity, ethical oversight, regulatory compliance, organizational readiness, and workforce preparedness as major determinants of successful AI adoption in healthcare [3,4,5,6,15,16,17,18,19,20,21,22,23,36,37,38,39,40]. Radiology professionals play a central role in this process by supervising AI-assisted clinical decision making, validating algorithm outputs, integrating AI into routine clinical workflows, and ensuring patient safety. Their knowledge, attitudes, and perceptions are therefore relevant to responsible implementation, but they cannot establish clinical or sustainability outcomes.

Survey-based literature already documents generally favorable attitudes toward AI together with concerns about education, professional roles, job displacement, clinical exposure, and organizational preparation. International studies of UK and Nordic radiography workforces have emphasized training deficits, professional identity, cultural change, and preservation of the human elements of care [35,41]. Saudi surveys have similarly examined AI knowledge, acceptance, education, clinical use, cybersecurity, workload, and professional-role expectations [42,43,44,45,46]. Although several of these studies used multidomain questionnaires, their primary objectives centered on attitudes, familiarity, education, professional roles, or culture change. Less attention has been given to jointly analyzing whether knowledge and favorable attitudes correspond to perceived institutional implementation capacity while concrete barriers are modeled concurrently across a mixed professional workforce.

Accordingly, this survey evaluated AI knowledge, attitudes, perceived institutional readiness, and implementation barriers, as well as their interrelationships, among radiology professionals in Saudi Arabia. The scientific contribution does not rest on being another national perception survey or on adding sustainability terminology. Rather, it lies in an integrated sociotechnical assessment of four linked constructs within the same 30-item instrument and across a mixed radiology workforce. The analysis tests a transferable implementation proposition: favorable attitudes may coexist with limited organizational capacity, and greater AI engagement may be associated with stronger recognition of implementation barriers. This acceptance–preparedness distinction is relevant beyond the Saudi setting because it discourages the use of positive attitudes as a proxy for operational readiness. Saudi Arabia provides a timely empirical setting for examining this proposition because of its ongoing digital-health transformation [32,33,34]. Sustainability is treated as an implementation context involving responsible resource use, workforce capacity, governance, and organizational resilience, not as the source of novelty or as a directly measured clinical or environmental outcome.

**Figure 1:**
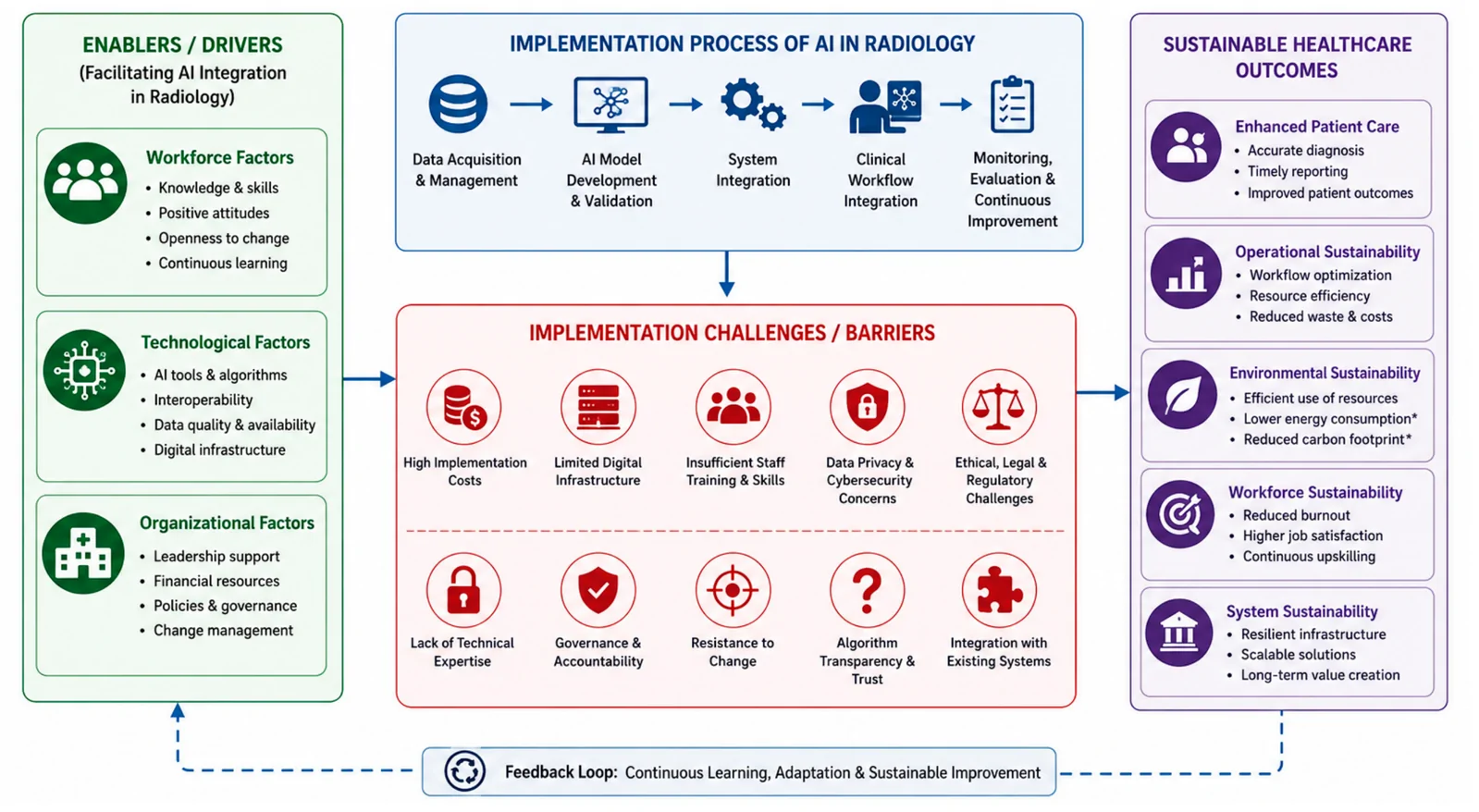
Conceptual framework developed from the literature review and study objectives, showing workforce factors, organizational capacity, implementation barriers, AI integration processes, and possible downstream healthcare outcomes. The outcome pathways shown on the right are literature-derived hypotheses and were neither measured nor tested in this survey. Only the eight barrier items Q23–Q30 listed in Supplementary Table S2 were administered. * indicates potential benefits that depend on implementation strategies and energy sources.

Based on the study’s objectives, we hypothesized that (H1) greater knowledge of AI would be positively associated with more favorable attitudes toward AI integration; (H2) positive attitudes would be associated with higher implementation readiness; and (H3) perceived implementation barriers would be negatively associated with organizational readiness for AI adoption (Figure 1).

## 2. Materials and Methods

### 2.1. Study Design and Setting

This study used a cross-sectional survey design to evaluate workforce readiness, organizational capacity, and implementation challenges associated with AI integration in radiology practice. The research was conducted in healthcare institutions across Saudi Arabia, focusing on healthcare professionals involved in radiology services, imaging operations, healthcare administration, medical physics, and AI-supported clinical activities. The cross-sectional approach was chosen for its effectiveness in evaluating perceptions, implementation readiness, and organizational barriers among various professional groups within a specific timeframe. The study was situated within the context of digital healthcare transformation and focused on factors influencing responsible AI integration rather than on measuring sustainability outcomes.

Sustainability was included only as a conceptual context for considering responsible implementation. The questionnaire assessed perceived workforce readiness, organizational capacity, governance, and implementation barriers; it did not directly measure environmental, economic, clinical, or broader sustainability outcomes. Accordingly, no sustainability outcome variable was included in the statistical analyses, and the findings cannot establish sustainability benefits or harms.

The study was conducted between February and April 2026 using an online self-administered questionnaire. Reporting followed the Strengthening the Reporting of Observational Studies in Epidemiology (STROBE) guidelines for cross-sectional studies (Supplementary Table S4).

### 2.2. Study Population and Participant Recruitment

The target population comprised healthcare professionals working in radiology-related services in Saudi Arabia, including radiologists, radiologic technologists, medical physicists, healthcare administrators, and professionals involved in artificial intelligence or healthcare data science. To be eligible for participation, respondents were required to

- Be currently practicing or employed in a radiology-related healthcare setting;
- Possess familiarity with radiology workflows, imaging services, or healthcare technologies;
- Provide voluntary informed consent.

The exclusion criteria included the following:

- Students and trainees without professional responsibilities;
- Incomplete questionnaire submissions;
- Duplicate responses;
- Individuals without professional involvement in radiology-related services.

Participants were recruited using a non-probability convenience sampling strategy. No comprehensive sampling frame of eligible radiology professionals or participating institutions was constructed. Between February and April 2026, the questionnaire link was distributed through institutional mailing lists, professional radiology societies, professional WhatsApp groups, LinkedIn professional networks, and email invitations. Because the channels overlapped and the number of unique eligible professionals who received or viewed the invitation was unknown, the number invited and a response rate could not be calculated. Participation was voluntary, anonymous, and uncompensated. To improve data integrity, the survey platform accepted only one response per device, and duplicate entries identified during data cleaning were removed before analysis.

Because selection probabilities were unknown and no sampling weights could be applied, the study was not designed to produce nationally representative estimates. The regional and professional composition of the sample reflects the reach of the distribution networks and respondents’ decisions to participate; it should not be interpreted as the distribution of the Saudi radiology workforce. The findings therefore describe the participating respondents and may be transferable only to professionals and institutions with similar characteristics and access to the recruitment channels.

### 2.3. Survey Instrument Development and Validation

The four domains were specified a priori rather than generated solely from the study data. Their selection reflected a sociotechnical view of AI adoption and evidence from AI-implementation guidance and previous healthcare-professional surveys [3,4,5,6,15,17,18,19,20,21,22,23,35,36,37,38,39,40,41,42,43,44,45,46]. The knowledge domain (Q1–Q7) captures familiarity with AI concepts, applications, limitations, and ethical considerations. The attitudes domain (Q8–Q14) captures perceived usefulness, expected clinical and workflow value, and willingness to support adoption. Implementation readiness (Q15–Q22) represents individual and organizational capacity, including infrastructure, training, policies, workflow integration, technical support, leadership, and personal preparedness. Perceived barriers (Q23–Q30) captures financial, infrastructural, workforce, technical, data-governance, ethical, legal, integration, and regulatory constraints. Separating these domains permits examination of whether professional knowledge and acceptance correspond to perceived operational capacity and implementation constraints.

An initial item pool was drafted from the same literature and organized to cover each prespecified domain. Ten experts in radiology, medical imaging, medical physics, and health research methodology reviewed the items for clarity, relevance, comprehensiveness, and alignment with the study objectives. Their qualitative recommendations were incorporated before pilot testing; a numerical item-level or scale-level content validity index was not calculated. The revised questionnaire was piloted with 30 radiology professionals (10 radiologists, 12 radiologic technologists, 5 medical physicists, and 3 healthcare administrators) to assess comprehensibility, completion time, the usability of response options, and ambiguous wording. Pilot feedback led to minor wording and formatting changes. Pilot participants were excluded from the final analysis.

The final instrument contained 30 items, and its complete wording and domain allocation are provided in Supplementary Table S2. It was administered in English, the language commonly used in medical education and professional communication among radiology professionals in Saudi Arabia. The final analysis did not use exploratory factor analysis to derive latent dimensions: the four domains were defined a priori, and their scores were calculated from the prespecified item allocations. Therefore, no extraction method, rotation, factor-retention rule, loading threshold, eigenvalue, or percentage of variance explained forms part of the final model. Construct-related evidence was limited to internal consistency and theoretically expected interdomain relationships. Confirmatory factor analysis, external convergent or discriminant validation, test–retest reliability, measurement invariance, and independent-sample cross-validation were not performed. The four-domain structure should therefore be regarded as theoretically justified and provisionally supported, not independently confirmed.

All items were evaluated using a five-point Likert scale:

- 1 = Strongly disagree;
- 2 = Disagree;
- 3 = Neutral;
- 4 = Agree;
- 5 = Strongly agree.

The Perceived Barriers and Implementation Challenges domain comprised eight items (Q23–Q30), addressing high implementation costs, limited digital infrastructure, insufficient staff training, lack of technical expertise, data privacy and security concerns, ethical and legal uncertainties, integration with existing systems, and lack of clear governance and regulatory frameworks. These eight items were the only barrier items administered and included in the barrier-domain analyses; no additional B-coded items formed part of the questionnaire.

### 2.4. Reliability and Construct-Related Evidence

Internal consistency was evaluated using Cronbach’s alpha for each prespecified domain and for the complete instrument. Cronbach’s alpha assesses the homogeneity of item responses but does not establish dimensionality or construct validity. The complete instrument yielded a Cronbach’s alpha coefficient of 0.89.

The domain-specific reliability coefficients were as follows:

- Knowledge: α = 0.89;
- Attitudes: α = 0.86;
- Implementation Readiness: α = 0.80;
- Perceived Barriers: α = 0.76.

All coefficients exceeded 0.70, supporting acceptable-to-good internal consistency in this sample. These coefficients should not be interpreted as confirmation of the four-domain factor structure, temporal stability, or validity across different professional or national populations.

### 2.5. Ethical Considerations

The study was conducted following the ethical PCAs outlined in the Declaration of Helsinki and the relevant institutional research governance requirements. Ethical approval was obtained from the Research Ethics Committee (MUREC) of Majmaah University, Saudi Arabia (Institutional Registration No. HA-01-R-088, approval no. MUREC-Feb.16/COM-2026/362).

Prior to participation, all respondents received an electronic information sheet describing the following:

- The objectives of the study;
- Voluntary participation requirements;
- Confidentiality safeguards;
- Data protection procedures;
- The right to withdraw at any time without penalty.

Informed consent was obtained from all participants before they were granted access to the questionnaire. No personally identifiable information was collected, and all responses were anonymized before analysis.

### 2.6. Statistical Analysis

Statistical analyses were performed using IBM SPSS Statistics version 30.0 (IBM Corp., Armonk, NY, USA). Descriptive statistics were used to summarize the participant characteristics and questionnaire responses. Continuous variables are presented as means ± standard deviations (SDs), whereas categorical variables are reported as frequencies and percentages. Questionnaires with incomplete responses were excluded during data cleaning; therefore, statistical analyses were performed using complete-case analysis.

The normality of the questionnaire domain scores was assessed using the Shapiro–Wilk test. Several variables significantly departed from normality (*p* < 0.05), supporting the use of nonparametric statistical methods. The differences among professional groups were evaluated using the Kruskal–Wallis H test because of the ordinal nature of the Likert-scale data and the observed non-normal distributions.

The associations among the principal questionnaire domains were examined using Spearman’s rank correlation coefficient. Internal consistency reliability was assessed using Cronbach’s alpha coefficients. Only the eight administered barrier items (Q23–Q30) were included in the barrier-domain analyses. All statistical tests were two-tailed, and statistical significance was established at *p* < 0.05. Analyses were descriptive and bivariate. No multivariable regression model was prespecified or performed; consequently, associations with demographic or professional characteristics were not interpreted as independent predictors.

### 2.7. Bias Reduction

Several measures were implemented to minimize potential sources of bias throughout the study. Participation was voluntary and anonymous, thereby reducing the likelihood of social desirability bias. Duplicate submissions and incomplete questionnaires were identified and removed during the data-cleaning process to improve data quality. In addition, standardized electronic data collection and a structured questionnaire minimized interviewer bias and ensured consistency in data acquisition.

Despite these measures, important biases remain. Convenience sampling and voluntary participation may have preferentially attracted professionals with greater interest in AI, stronger opinions about implementation, greater digital literacy, or closer connections to the distributing networks. This self-selection could overestimate AI knowledge or favorable attitudes and could also alter estimates of perceived barriers. Exclusive electronic distribution may have under-represented professionals with limited digital access or lower engagement with online professional platforms. In addition, the absence of a sampling frame and a known invitation denominator prevented calculation of a response rate. Characteristics of nonrespondents were unavailable, so respondents and nonrespondents could not be compared and nonresponse bias could not be quantified. Device-level duplicate controls improved response integrity but did not address coverage, self-selection, or nonresponse bias.

### 2.8. Clinical Trial Registration

Clinical trial number: not applicable.

This study was observational and did not include clinical intervention, patient randomization, or experimental treatment allocation. Therefore, clinical trial registration was not necessary.

## 3. Results

### 3.1. Participant Characteristics

A total of 295 valid questionnaires were included in the final analysis after the removal of incomplete and duplicate responses. Among the participants, 203 (68.8%) were male and 92 (31.2%) were female. Radiologists constituted the largest professional category (n = 97, 32.9%), followed by radiographers (n = 89, 30.2%). Medical physicists accounted for 36 participants (12.2%), while healthcare administrators and AI/data science professionals represented 29 participants (9.8%). Other radiology-related professionals comprised 15 (5.1%) participants. The largest subgroup had 5–10 years (34.2%), followed by those with less than 5 years (25.8%), 11–15 years (20.3%), and more than 15 years (19.7%). Educational attainment was predominantly at the bachelor’s degree level (53.2%), followed by master’s degree holders (34.6%), professional certification holders (8.1%), and doctorate-level participants (4.1%). Participants were recruited from all major regions of Saudi Arabia, with the highest representation originating from the Central Region (42.4%), followed by the Western Region (21.4%), Eastern Region (17.3%), Southern Region (11.5%), and Northern Region (7.4%) (Table 1) (Supplementary Table S1).

### 3.2. Principal Study Domain Scores

Table 2 presents the mean scores across the four questionnaire domains. Perceived barriers had the highest mean score (3.67 ± 0.64), followed by attitudes (3.57 ± 0.69), knowledge (3.35 ± 0.70), and implementation readiness (3.04 ± 0.79). Detailed item-level descriptive statistics are provided in Supplementary Table S3.

### 3.3. Barrier Analysis

Scores for the eight administered barrier items (Q23–Q30) ranged from 3.64 to 3.98. High implementation costs had the highest mean score (3.98 ± 0.71), followed by limited digital infrastructure (3.90 ± 0.75), insufficient staff training (3.84 ± 0.77), and lack of technical expertise (3.82 ± 0.78). The complete eight-item barrier analysis is presented in Table 3. Shapiro–Wilk tests indicated significant deviations from normality for all barrier variables (*p* < 0.001).

### 3.4. Reliability Analysis

Cronbach’s alpha coefficients were 0.89 for knowledge, 0.86 for attitudes, 0.80 for implementation readiness, and 0.76 for perceived barriers; the overall instrument coefficient was 0.89 (Table 4). These estimates apply to the prespecified composite domains. No latent factor solution was retained; therefore, no eigenvalues or total-variance-explained percentage is reported.

### 3.5. Correlation Analysis

Spearman correlation analysis showed positive correlations between knowledge and attitudes (r = 0.49), knowledge and implementation readiness (r = 0.39), knowledge and perceived barriers (r = 0.29), attitudes and implementation readiness (r = 0.44), attitudes and perceived barriers (r = 0.25), and implementation readiness and perceived barriers (r = 0.38). The complete correlation matrix is presented in Table 5.

**Figure 2:**
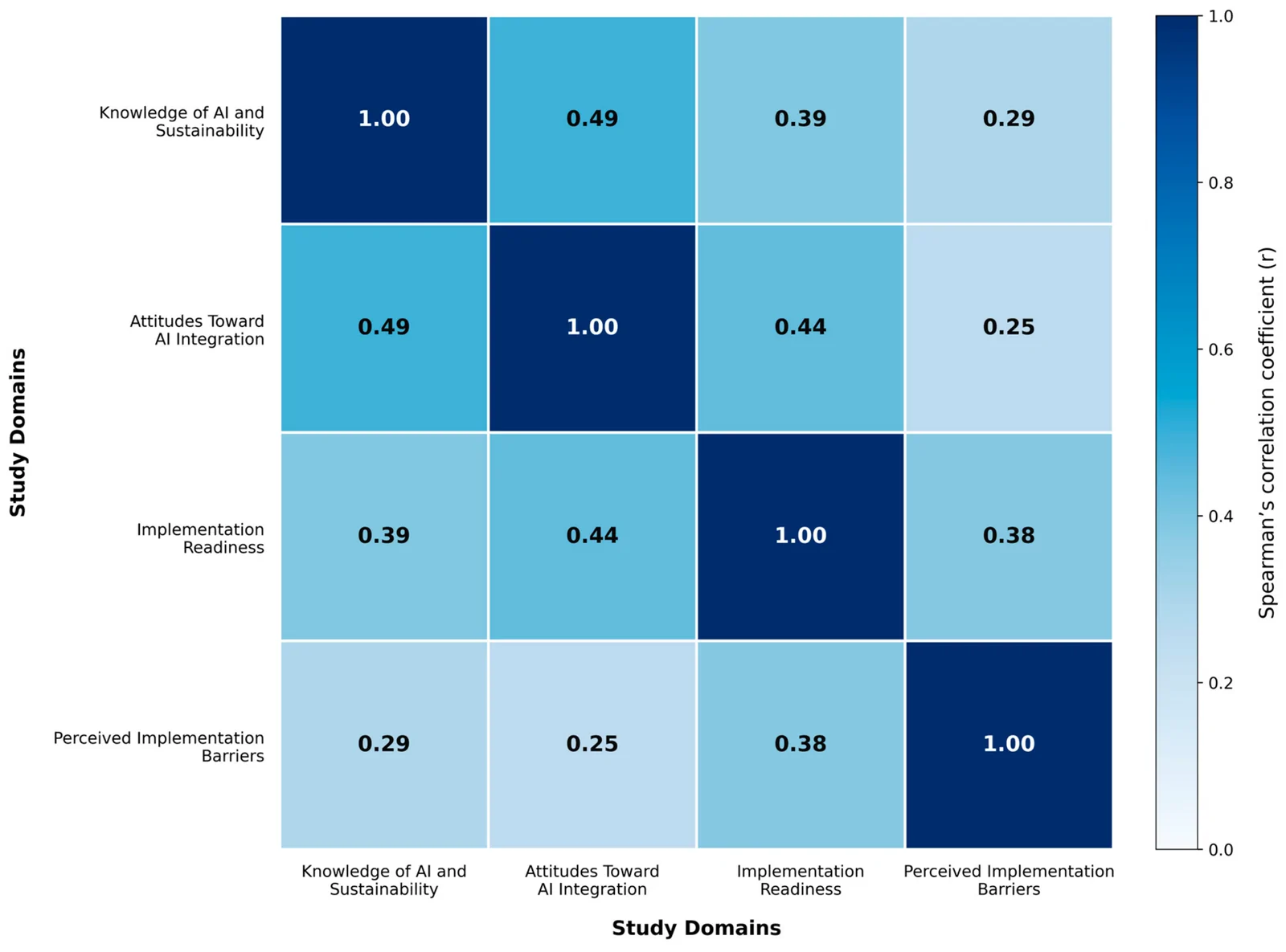
Heatmap of Spearman correlation matrix between major study domains (n = 295).

The coefficients reported in Figure 2 and Table 5 are reproduced in Supplementary Figure S1 as an alternative network visualization.

### 3.6. Unadjusted Professional Group Comparisons

Kruskal–Wallis tests identified no statistically significant differences among the professional groups across the principal questionnaire domains (all *p* > 0.05). Domain scores for each professional group are presented in Table 6. These comparisons were unadjusted and do not identify independent demographic or professional predictors of implementation readiness or perceived barriers.

## 4. Discussion

The principal scientific contribution is the integrated cross-domain analysis rather than a national estimate of favorable AI perceptions. Earlier surveys have generated important evidence on attitudes, education, professional identity, and implementation concerns [35,41,42,43,44,45,46], whereas the present study assesses knowledge, attitudes, perceived institutional readiness, and implementation barriers concurrently and examines their interrelationships across multiple radiology-related professions. This design reveals an acceptance–preparedness divergence: favorable views of AI do not establish that departments possess the infrastructure, governance, financing, technical support, and workforce capacity required for routine adoption. This distinction provides a transferable implementation hypothesis for other health systems and explains why workforce acceptance should not be used as a surrogate for organizational readiness. Because the data are cross-sectional, self-reported, and convenience-sampled, this contribution is conceptual and hypothesis-generating rather than evidence of national prevalence or causality.

Saudi surveys provide related but not identical findings. Alelyani et al. reported favorable attitudes and support for including AI in health-professions curricula [41]. However, Qurashi et al. found that 82% of radiology personnel had never used AI in their departments and 71.4% had received no formal AI education [42], while Hamd et al. reported limited knowledge or inadequate training among 58.6% of respondents [45]. The present study extends beyond education by identifying financing, infrastructure, technical capacity, and governance concerns. These differences may reflect the instruments, recruitment methods, professional samples, and timing rather than a simple progression in readiness. Training needs are recurrent, but the studies do not establish that training alone is sufficient for implementation or permit direct numerical comparison.

Barrier priorities also differ within the Saudi literature. Alyami et al. emphasized limited knowledge and cybersecurity [44], whereas respondents in the present study prioritized financial cost and digital infrastructure. Khafaji et al. reported concerns about employment and workload among radiology residents [43], issues that were less prominent in this mixed-profession sample. The absence of statistically significant professional-group differences in the present study contrasts with role-related differences reported previously [42,43]. Nevertheless, unequal and sometimes small subgroups, different instruments, and differing professional compositions limit this comparison; the null result should not be interpreted as evidence that all professions have identical implementation or training needs.

International studies do not reproduce the same barrier hierarchy. In the United Kingdom R-AI-diographers survey, 60.7% of respondents were optimistic about the medium-to-long-term effects of AI and 81.2% did not expect replacement by AI; nevertheless, concerns centered on deskilling and AI inefficiencies, and 70.8% had received no formal AI training [35]. In contrast, the present study ranked costs, digital infrastructure, training, and technical expertise as the leading barriers, while job displacement and professional identity were not measured separately. The Nordic study framed readiness as a radiography-specific culture-change issue [46], whereas the present instrument emphasized institutional capacity and governance. These differences question a universal barrier profile but cannot be attributed confidently to national context: the UK study included radiographers and students recruited through snowball sampling, the Nordic study examined a radiography-specific workforce, and the present convenience sample combined several professions. Differences in instruments, outcomes, and scoring also prevent direct numerical comparison. Thus, shared optimism and preparedness concerns support only a broad hypothesis, not equivalence or external confirmation across healthcare systems.

The observed associations also require cautious interpretation. The relationships of greater AI knowledge with favorable attitudes and perceived readiness are consistent with Saudi survey evidence linking education and familiarity to engagement with AI [42,45]. However, the UK and Nordic studies did not assess the same four-domain correlation structure [35,46]; their findings therefore cannot be treated as external confirmation of the present associations. The positive relationships involving perceived barriers may indicate that more informed or better-prepared professionals recognize implementation constraints more clearly, rather than that they are more resistant to adoption. The reliability coefficients support internal consistency of the questionnaire scores in this sample, but they do not establish objective AI competence, institutional readiness, or the factorial validity of the four-domain structure. The cross-sectional design also prevents determination of the direction of these relationships.

### 4.1. Conceptual Relevance to Sustainable Healthcare

The relevance of these findings to sustainable healthcare is conceptual. The survey identifies perceived conditions that may support responsible implementation, but it does not show that AI improves service efficiency, diagnostic capacity, resource use, access, or organizational resilience. Such potential benefits should be treated as hypotheses for evaluation rather than demonstrated consequences of AI adoption. Institutions should assess these outcomes directly and determine whether proposed applications introduce financial, data-governance, or environmental burdens.

### 4.2. Environmental Sustainability as a Future Evaluation Domain

No environmental sustainability variable was included in this study. Future evaluations may examine whether workflow optimization reduces unnecessary imaging, repeated examinations, or inefficient equipment use while accounting for the energy consumed by model development, deployment, data storage, and computation [7,8,9,10,16]. Relevant indicators include energy consumption, computational demand, equipment utilization, repeat-examination rates, data-storage requirements, and electricity carbon intensity. Environmental benefit must be demonstrated through operational and life-cycle assessment rather than inferred from workforce perceptions or perceived technological efficiency.

### 4.3. Study Limitations

Finally, while the authors have acknowledged several limitations, additional discussion is warranted regarding the reliance on self-reported perceptions, the cross-sectional design that precludes causal inference, the absence of objective organizational readiness measures, and the potential influence of social desirability bias. Self-reported perceptions may not accurately reflect objective AI competency, institutional infrastructure, or governance capacity. Future research should complement perceptual surveys with objective assessments, including competency testing, infrastructure audits, and direct observation of AI integration practices. Furthermore, the cross-sectional nature of this study prevents determination of whether knowledge and attitudes precede or follow perceptions of readiness, or whether these relationships are bidirectional. Longitudinal and implementation-based designs are needed to establish temporal sequences and causal pathways. The absence of objective organizational readiness measures—such as documented governance policies, available technical infrastructure, budget allocations, or training program evaluations—limits our ability to verify whether perceived readiness corresponds to actual institutional capacity. Additionally, while voluntary and anonymous participation was intended to minimize social desirability bias, the possibility remains may have provided responses they perceived as favorable or aligned with institutional expectations. The self-selected nature of the sample may also have attracted professionals with stronger opinions about AI, potentially overestimating both favorable attitudes and recognition of barriers.

### 4.4. Evidence-Informed Recommendations

Current and previous survey evidence supports a phased implementation strategy that combines professional development with organizational preparation. Healthcare institutions should establish multidisciplinary AI governance, assess infrastructure, interoperability, cybersecurity, and data quality, provide role-specific education, and introduce selected applications through monitored pilot programs. Evaluations should measure diagnostic performance, workflow effects, patient safety, user experience, economic value, resource use, and environmental impact.

Prospective studies are needed to determine whether particular combinations of education, governance, infrastructure investment, and workflow redesign produce safe and effective improvements and independently verifiable sustainability benefits in Saudi radiology services. Given the descriptive and unadjusted design, these recommendations represent provisional, hypothesis-generating priorities rather than effects established through adjusted analysis.

## 5. Conclusions

This study assessed radiology professionals’ knowledge, attitudes, perceived institutional readiness, and perceived barriers regarding AI integration. Participants reported favorable attitudes and moderate knowledge, whereas perceived readiness was lower and organizational, infrastructural, training, and governance barriers remained prominent. These findings support a sociotechnical approach that combines workforce development with organizational preparation, infrastructure, governance, and ongoing evaluation. They do not demonstrate improvements in healthcare quality, efficiency, patient outcomes, progress toward the Sustainable Development Goals, or environmental sustainability because these outcomes were not measured. Sustainability should therefore be regarded as a conceptual implementation context and a priority for future evaluation, not as an evidence-based outcome of this survey. Prospective implementation studies should directly measure clinical performance, workflow, economic value, resource use, and environmental effects over time.

## Figures and Tables

**Table 1:** Demographic and Professional Characteristics of Participants (n = 295).

Variable	Category	n (%)
Gender	Male	203 (68.8)
	Female	92 (31.2)
Professional Role	Radiologist	97 (32.9)
	Radiologic Technologist	89 (30.2)
	Medical Physicist	36 (12.2)
	Healthcare Administrator	29 (9.8)
	AI/Data Scientist	29 (9.8)
	Other	15 (5.1)
Years of Experience	<5 years	76 (25.8)
	5–10 years	101 (34.2)
	11–15 years	60 (20.3)
	>15 years	58 (19.7)
Education Level	Bachelor’s degree	157 (53.2)
	Master’s degree	102 (34.6)
	Professional certification	24 (8.1)
	Doctorate	12 (4.1)

**Table 2:** Mean Scores Across Principal Study Domains.

Domain	Mean ± SD
Knowledge	3.35 ± 0.70
Attitudes	3.57 ± 0.69
Implementation Readiness	3.04 ± 0.79
Perceived Barriers	3.67 ± 0.64

**Table 3:** Descriptive Analysis of the Eight Administered Barrier Items (Q23–Q30).

Barrier	Mean ± SD	Median	Shapiro–Wilk *p*
High implementation costs	3.98 ± 0.71	4.0	<0.001
Limited digital infrastructure	3.90 ± 0.75	4.0	<0.001
Insufficient staff training	3.84 ± 0.77	4.0	<0.001
Lack of technical expertise	3.82 ± 0.78	4.0	<0.001
Data privacy and cybersecurity concerns	3.75 ± 0.76	4.0	<0.001
Ethical and legal accountability concerns	3.69 ± 0.80	4.0	<0.001
System integration challenges	3.64 ± 0.82	4.0	<0.001
Governance and regulatory uncertainty	3.66 ± 0.78	4.0	<0.001

**Table 4:** Reliability Assessment.

Domain	Cronbach’s α
Knowledge	0.89
Attitudes	0.86
Implementation Readiness	0.80
Perceived Barriers	0.76
Overall Instrument	0.89

**Table 5:** Spearman Correlation Matrix.

Domain	Knowledge	Attitudes	Implementation Readiness	Barriers
Knowledge	1.00	0.49	0.39	0.29
Attitudes	0.49	1.00	0.44	0.25
Implementation Readiness	0.39	0.44	1.00	0.38
Barriers	0.29	0.25	0.38	1.00

**Table 6:** Domain Scores Across Professional Groups.

Professional Group	Knowledge	Attitudes	Readiness	Barriers
AI/Data Scientist	3.33	3.43	2.97	3.59
Healthcare Administrator	3.33	3.54	2.91	3.69
Medical Physicist	3.39	3.44	3.13	3.63
Other	3.23	3.62	2.99	3.53
Radiologic Technologist	3.41	3.63	3.07	3.75
Radiologist	3.31	3.61	3.05	3.67

## Data Availability

The datasets generated and/or analyzed during the current study are available from the corresponding author upon reasonable request, subject to institutional ethical and data governance requirements.

## Supplementary Materials

The following supporting information can be downloaded at https://www.mdpi.com/article/10.3390/healthcare14172711/s1: Supplementary Table S1 (regional distribution); Supplementary Table S2 (questionnaire items and domain allocation); Supplementary Table S3 (item-level descriptive statistics); and Supplementary Table S4 (STROBE checklist); Supplementary Figure S1 (correlation network of the four study domains).

## Abbreviations

The following abbreviations are used in this manuscript:

AI	Artificial Intelligence
SDGs	Sustainable Development Goals
STROBE	Strengthening the Reporting of Observational Studies in Epidemiology
SD	Standard Deviation
PACS	Picture Archiving and Communication Systems
RIS	Radiology Information Systems
