# Supplementary material for: Artificial Intelligence Integration in Radiology in Saudi Arabia: A Cross-Sectional Survey of Workforce Readiness and Implementation Challenges
Source: Healthcare (Basel). 2026 Aug 25;14(17):2711. doi: 10.3390/healthcare14172711 (PMC13564685; doi:10.3390/healthcare14172711)

Supplementary Materials

Artificial Intelligence Integration in Radiology in Saudi Arabia: A Cross-Sectional Survey of Workforce Readiness and Implementation Challenges

This file contains Supplementary Tables S1–S4 and Supplementary Figure S1. The supplementary materials correspond to the prespecified four-domain analysis reported in the manuscript.

Supplementary Table S1. Geographical distribution of participants across the five major administrative regions of Saudi Arabia (n = 295). Participants were recruited from healthcare institutions and professional networks.

Region	Number of participants (n)	Percentage (%)
Central Region (Riyadh, Qassim, Majmaah and surrounding areas)	125	42.4
Western Region (Makkah, Madinah, Jeddah, Taif)	63	21.4
Eastern Region (Dammam, Khobar, Jubail and surrounding areas)	51	17.3
Southern Region (Asir, Jazan, Najran, Al-Baha)	34	11.5
Northern Region (Tabuk, Hail, Al-Jouf, Northern Borders)	22	7.4
Total	295	100.0

Supplementary Table S2. Questionnaire items and domain allocation used to assess perceptions of AI integration in radiology.

Domain	Item code	Questionnaire item
Knowledge of AI and sustainability (Q1–Q7)	Q1	I have a good understanding of artificial intelligence concepts used in radiology.
	Q2	I am familiar with current AI applications in medical imaging.
	Q3	I understand how AI can contribute to healthcare sustainability.
	Q4	I am aware of AI-assisted diagnostic tools used in radiology practice.
	Q5	I understand the benefits and limitations of AI systems.
	Q6	I am familiar with ethical considerations related to AI in healthcare.
	Q7	I have sufficient knowledge to work with AI-enabled radiology systems.
Attitudes toward AI and sustainability (Q8–Q14)	Q8	AI can improve diagnostic accuracy in radiology.
	Q9	AI can enhance workflow efficiency in imaging departments.
	Q10	AI can support more sustainable healthcare delivery.
	Q11	AI should be integrated into routine radiology practice.
	Q12	AI can reduce unnecessary imaging examinations.
	Q13	AI can improve patient outcomes and service quality.
	Q14	I support broader implementation of AI technologies in radiology.
Implementation readiness and practice (Q15–Q22)	Q15	My institution is prepared for AI implementation in radiology services.
	Q16	Adequate digital infrastructure exists to support AI deployment.
	Q17	Staff members receive sufficient training regarding AI applications.
	Q18	AI-related policies and procedures are available within my institution.
	Q19	Radiology workflows can be effectively integrated with AI systems.
	Q20	Technical support is available for AI implementation and maintenance.
	Q21	Leadership supports AI adoption initiatives.
	Q22	I feel prepared to use AI technologies in my professional practice.
Perceived barriers and challenges (Q23–Q30)	Q23	High implementation costs limit AI adoption.
	Q24	Limited digital infrastructure represents a major obstacle.
	Q25	Insufficient staff training hinders AI implementation.
	Q26	Lack of technical expertise is a barrier to adoption.
	Q27	Data privacy and security concerns impede implementation.
	Q28	Ethical and legal uncertainties affect AI acceptance.
	Q29	Integration with existing systems is challenging.
	Q30	Lack of clear governance and regulatory frameworks limits AI adoption.

Supplementary Table S3. Item-level descriptive statistics for all questionnaire items (n = 295). Responses were measured using a five-point Likert scale ranging from 1 (strongly disagree) to 5 (strongly agree).

Item	Questionnaire statement (abbreviated)	Mean ± SD
Q1	Understanding of AI concepts in radiology	3.36 ± 0.87
Q2	Familiarity with AI applications in medical imaging	3.42 ± 0.85
Q3	Understanding AI contribution to healthcare sustainability	3.35 ± 0.91
Q4	Awareness of AI-assisted diagnostic tools	3.38 ± 0.82
Q5	Understanding benefits and limitations of AI	3.44 ± 0.79
Q6	Familiarity with ethical aspects of AI	3.29 ± 0.86
Q7	Knowledge sufficient for AI-enabled practice	3.23 ± 0.90
Q8	AI improves diagnostic accuracy	3.55 ± 0.91
Q9	AI enhances workflow efficiency	3.68 ± 0.83
Q10	AI supports healthcare sustainability	3.61 ± 0.86
Q11	AI should be integrated into radiology practice	3.59 ± 0.88
Q12	AI reduces unnecessary imaging	3.54 ± 0.99
Q13	AI improves patient outcomes	3.58 ± 0.87
Q14	Support for broader AI implementation	3.46 ± 0.92
Q15	Institutional readiness for AI implementation	3.11 ± 1.06
Q16	Availability of digital infrastructure	2.97 ± 1.15
Q17	Availability of AI training programs	3.02 ± 1.11
Q18	Existence of AI policies and procedures	3.06 ± 1.09
Q19	Integration of AI into existing workflows	3.09 ± 1.12
Q20	Availability of technical support	3.03 ± 1.18
Q21	Leadership support for AI adoption	3.05 ± 1.10
Q22	Personal readiness to use AI tools	3.00 ± 1.13
Q23	High implementation costs	3.98 ± 0.71
Q24	Limited digital infrastructure	3.90 ± 0.75
Q25	Insufficient staff training	3.84 ± 0.77
Q26	Lack of technical expertise	3.82 ± 0.78
Q27	Data privacy and cybersecurity concerns	3.75 ± 0.76
Q28	Ethical and legal accountability concerns	3.69 ± 0.80
Q29	System integration challenges	3.64 ± 0.82
Q30	Lack of governance and regulatory clarity	3.66 ± 0.78

Supplementary Table S4. STROBE checklist for the cross-sectional survey.

Manuscript title: Artificial Intelligence Integration in Radiology in Saudi Arabia: A Cross-Sectional Survey of Workforce Readiness and Implementation Challenges

STROBE item	Recommendation	Section/page
Title and abstract		
1(a)	Indicate the study design in the title or abstract	Title; Abstract
1(b)	Provide an informative and balanced summary	Abstract
Introduction		
2	Explain scientific background and rationale	Introduction
3	State specific objectives and hypotheses	End of Introduction
Methods		
4	Present key elements of study design	Section 2.1
5	Describe setting, locations, and relevant dates	Section 2.1
6(a)	Describe eligibility criteria and participant selection	Section 2.2
7	Define outcomes, exposures, predictors, and variables	Sections 2.3–2.7
8	Describe data sources and measurement methods	Section 2.3
9	Describe efforts to address bias	Sections 2.2, 5.3
10	Explain study size determination	Section 2.2
11	Explain handling of quantitative variables	Section 2.6
12(a)	Describe statistical methods	Section 2.6
12(b)	Describe methods used for subgroup analyses	Sections 2.6, 3.7
12(c)	Explain handling of missing data	Section 2.2
12(d)	Describe analytical methods accounting for sampling strategy	Section 2.6
12(e)	Describe sensitivity analyses (if applicable)	Not applicable
Results		
13(a)	Report participant numbers at each stage	Section 3.1
13(b)	Provide reasons for non-participation	Section 2.2
13(c)	Consider use of a flow diagram	Figure (optional)
14(a)	Describe participant characteristics	Table 1; Section 3.1
14(b)	Indicate missing data for variables	Section 2.2
15	Report outcome events or summary measures	Tables 2–8
16(a)	Present main results with estimates	Sections 3.2–3.7
16(b)	Report category boundaries if applicable	Section 2.3
16(c)	Translate estimates into meaningful interpretations	Sections 3.2–3.7
17	Report additional analyses	Sections 3.4–3.7
Discussion		
18	Summarize key findings	Section 4
19	Discuss limitations	Section 5.3

STROBE item	Recommendation	Section/page
20	Provide interpretation considering objectives and evidence	Sections 4 and 6
21	Discuss generalizability	Sections 5.3 and 6
Other information		
22	Report funding sources and role of funders	Funding Statement
23	Report ethical approval and consent procedures	Ethics Approval Statement

Study design: Cross-sectional survey   Reporting guideline: STROBE Statement for Observational Studies

Supplementary Figure S1. Spearman correlation network among the four study domains (n = 295). Edge labels reproduce the coefficients reported in Table 5 and Figure 2.

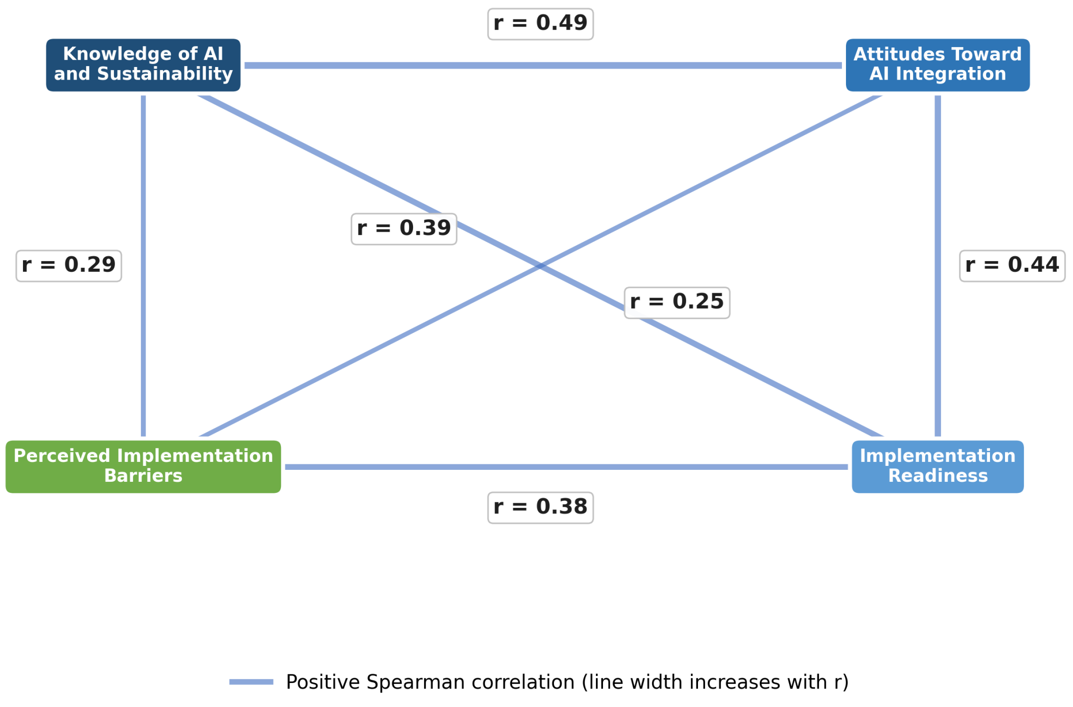

Supplement: Supplementary file 1 [file healthcare-14-02711-s001.zip › healthcare-4420819-supplementary.pdf]
